# Vogt-Koyanagi-Harada Disease: A Case Series in a Tertiary Eye Center

**DOI:** 10.1155/2021/8848659

**Published:** 2021-01-22

**Authors:** Pranav Shrestha, Sadhana Sharma, Ranju Kharel

**Affiliations:** ^1^Mechi Eye Hospital, Jhapa 57200, Nepal; ^2^Department of Uveitis, B. P. Koirala Lions Center for Ophthalmic Studies (BPKLCOS), Institute of Medicine, Maharajgunj, Kathmandu 44600, Nepal

## Abstract

**Purpose:**

To study the clinical spectrum of Vogt-Koyanagi-Harada (VKH) disease in a tertiary eye center in Nepal.

**Methods:**

Baseline demographic details and clinical features of six patients diagnosed as VKH in a tertiary eye center were retrospectively reviewed. Examinations included best corrected visual acuity, intraocular pressure, and slit lamp examination of anterior and posterior segments. Baseline blood investigations, chest X-ray, fundus photography, and fundus fluorescent angiography (FFA) were performed on all the patients.

**Results:**

The mean age at presentation was 46 ± 8.43 years with female predominance (83.3%). The disease was complete in 16.7%, incomplete in 16.7%, and probable in 66.7% of the patients. Most cases presented in acute uveitis stage (66.7%). The most common finding in acute cases was serous retinal detachment (66.7%), followed by disc edema (58.3%), whereas in chronic cases, depigmented fundus was the most common. All cases had bilateral presentation.

**Conclusion:**

VKH is an important cause of bilateral loss of vision and has a good visual prognosis if aggressive treatment is initiated during the early stages.

## 1. Introduction

Vogt-Koyanagi-Harada (VKH) is a severe, bilateral, granulomatous panuveitis associated with extraocular manifestations. It is a cell-mediated autoimmune disorder affecting the pigmented tissues leading to amplification of inflammatory cascade against the melanocytes [1, 2]. It is more common in the adult population and has a predilection for female gender. HLA-DQ4 carriers had a higher risk of VKH, and HLA-DQ1 seemed to be protective [3]. Most of the cases are bilateral; however, in rare cases, a unilateral presentation or delayed involvement of the other eye can also occur [4, 5].

Patients may present with varied ocular manifestations like iridocyclitis, diffuse choroidal thickening, and hyperemia of the optic disc. Headache and meningismus are the main neurological manifestations, while auditory and integumentary changes may present with tinnitus and hearing loss and alopecia, poliosis, and vitiligo, respectively [6].

The four distinct phases of VKH are as follows: prodromal, acute uveitic, convalescent, and chronic recurrent. Low-grade nongranulomatous anterior uveitis along with diffuse choroiditis and exudative retinal detachment (RD) is seen in acute phases, while in chronic recurrent phases, patients present with recurrent granulomatous anterior uveitis [1]. As the frequency of ocular inflammatory attacks increases, the occurrence of ocular complications increases and is associated with worse visual prognosis.

The first international workshop defined VKH as bilateral uveitis without a history of trauma or ocular surgery [2] and classified VKH disease as (a) complete VKH with ocular, neurological, or auditory and integumentary involvement; (b) incomplete VKH with ocular and either neurological/auditory or integumentary involvement; and (c) probable VKH without extraocular manifestation [4, 5, 7, 8].

Treatment regimens include corticosteroids (intravenous, oral, and regional) and immunomodulators. Improper and inadequate treatment during the acute stage can lead to chronic recurrent VKH disease. High-dose systemic corticosteroids initiated during the early acute uveitis stage followed by slow tapering is required to suppress the inflammation. Addition of immunomodulatory agents along with steroids can improve the visual outcome and can also decrease the risk of cutaneous involvement [1].

We present a series of 6 cases of VKH presenting to a tertiary eye center in Nepal over a period of one year.

## 2. Methods

This is a retrospective review of medical records of VKH patients presenting to our uveitis clinic from 2015 to 2017 with a follow-up of 1-3 years. Informed consent was obtained from all the patients, and the study adhered to the Declaration of Helsinki. All patients provided a detailed history and underwent ocular examination including visual acuity (VA), intraocular pressure (IOP), and slit lamp evaluation including fundus evaluation using 90D aspheric lens and indirect ophthalmoscopy using 20D aspheric lens. Ultrasound B scan was done when required, and patients were required to undergo fundus photography at various stages of treatment depending upon the visibility of the posterior segment. All the patients were subjected to chest X-ray and baseline blood investigation including complete blood count (CBC), erythrocyte sedimentation rate (ESR), C reactive protein (CRP), uric acid, random blood sugar (BSR), serology for HBsAg, HCV, HIV, and syphilis.

The diagnosis of VKH is made by the criteria as summarized in Table 1.

Due to the wide range of presentation, the disease is categorized as follows: probable or ocular VKH if all the ocular criteria mentioned in Table 1 are met, incomplete VKH if along with ocular criteria there is presence of integumentary or neurological criteria, and complete VKH if both integumentary and neurological criteria are present.

After preliminary diagnosis of VKH was made, the patients were referred for consultation with a neurophysician, otorhinolaryngologist, and dermatologist. Depending on their expertise, further investigation like CSF examination was done solely on their discretion.

## 3. Results

**Figure 1:**
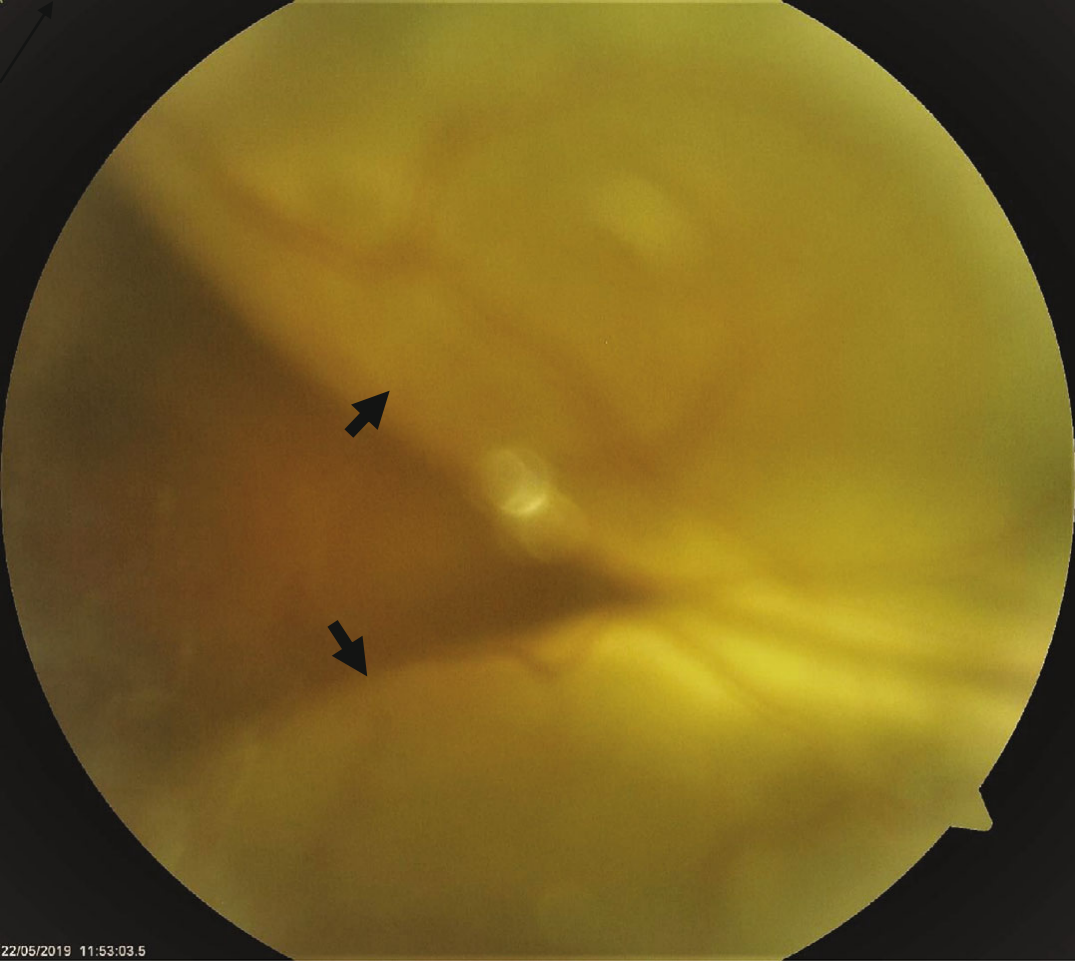
A VKH patient presenting in acute uveitic phase with bullous exudative retinal detachment (black arrows).

A total of 12 eyes of six patients of different ethnicities were evaluated. Among them, 5 (83.3%) were female. The mean age at presentation was 46 ± 8.43 years (range 30–54 years). Four out of six patients (66.7%) presented in acute uveitis phase (Figure 1), while one (16.7%) patient each presented in convalescent phase and chronic recurrent phase. Among the six cases, four (66.7%) were probable VKH, one (16.7%) was incomplete, and one (16.67%) was complete.

**Figure 2:**
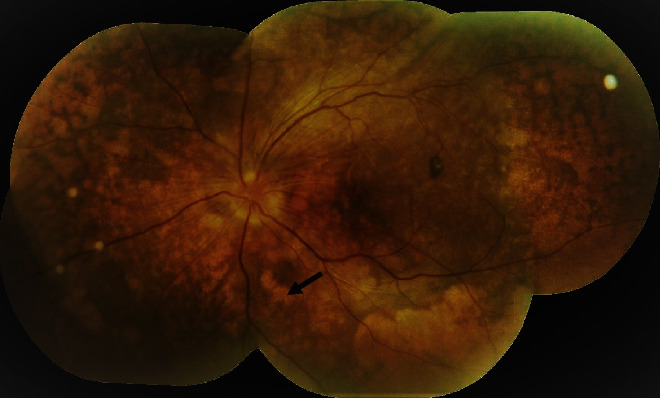
A VKH patient presenting in chronic stage with sunset glow fundus, multiple nummular chorioretinal depigmented scars, and pigmentary changes.

**Figure 3:**
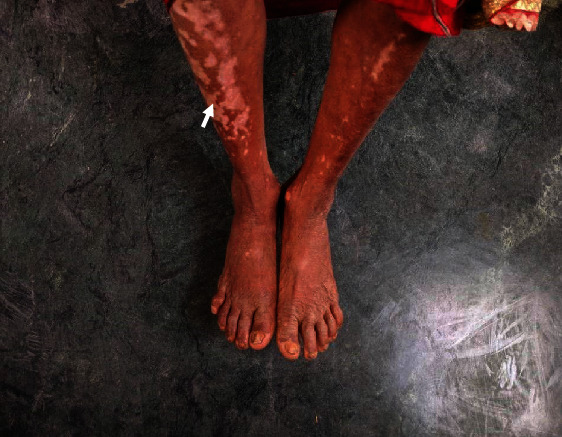
A VKH patient with extraocular manifestation (vitiligo).

The most common presenting symptom was diminution of vision in 91.7% (11 out of 12 eyes) and photophobia in 58.3% (7 out of 12 eyes). Majority of the eyes (58.3%; 7 out of 12) presented in the acute uveitis stage. Posterior segment findings included disc edema in 58.3% (7 out 12 eyes), exudative retinal detachment in 66.7% (8 out of 12 eyes), and choroiditis in 33.3% (4 out of 12 eyes). During the study duration, 6 eyes (50%) developed hypopigmented fundus with sunset glow appearance (Figure 2). Extraocular manifestation was present in two cases. Integumentary findings (vitiligo) (Figure 3) were present in both the cases, and one case also had neurological (headache) and auditory (tinnitus) findings.

**Figure 4:**
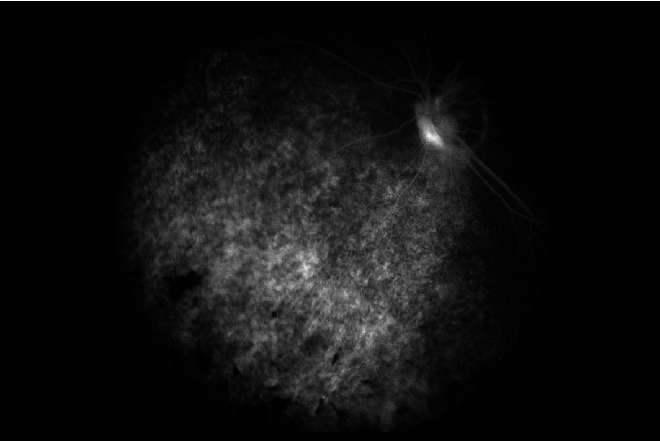
FFA showing multiple pinpoint hyperfluorescence in arteriovenous phase of the angiogram.

The fluorescent angiography (FA) findings in most of the eyes were multiple spots of pinpoint hyperfluorescence in the early arteriovenous phase (Figure 4) with pooling of the dye within the serous RD in the late phase of the angiogram.

At the first presentation, four out of six cases (66.7%) received oral and topical corticosteroids, while two cases (33.3%) received intravenous corticosteroids. Immunomodulator therapy either with azathioprine or methotrexate was given later to control recurrence in cases 2 and 3, respectively (Table 2). Two of the cases, in addition to the immunomodulator therapy, also received antitubercular treatment (ATT) for 9 months due to a mixed picture of the disease.

The most common complication was cataract, comprising 50% (6 out of 12 eyes), for which cataract surgery was done. Glaucoma filtration surgery was done in two eyes (33.3%) for secondary glaucoma not controlled with topical medications. All the patients in the series attained a final visual acuity of ≥6/24 (Table 1).

## 4. Discussion

Vogt-Koyanagi-Harada disease is relatively uncommon among the Nepalese population, and extraocular manifestation of the disease is even rare [2]. The prognosis in terms of vision remain good when proper treatment is provided even if there is presence of structural changes in the retinal outer layers [3].

The mean age and the female predominance in our series correlates with those presented in other literature. Disc hyperemia and serous retinal detachment were the most common posterior segment presentation similar to comparative literature [1]. Only few cases had extraocular manifestation in this series even though they presented with a typical fundus finding, the possibility of which has been explored in the study by Beniz et al. [9].

Optical coherence tomography could prove to be an invaluable imaging modality for early detection of macula-related complications and detection of the subretinal fluids [10]. In addition, if enhanced depth imaging (EDI) is available, the amount of choroidal inflammation could also be assessed depending on the stage of the disease [11].

Similar to the finding of Paredes et al. [12], immunomodulator therapy along with tapered systemic and topical application of a corticosteroid resulted in a better visual outcome. In this series, two cases were receiving antitubercular therapy along with an immunomodulator due to the possibility of the antigenic mimicry and cross-reactivity between the two conditions [13].

## 5. Conclusion

VKH is a multisystem disorder, and diagnosis is mainly clinical. The mainstay of treatment is aggressive corticosteroid therapy in the acute phase and an additional immunosuppressive agent. There is a potential for significant visual loss, but timely identification and treatment can minimize ocular morbidity.

## Figures and Tables

**Table 1:** Diagnostic criteria of VKH.

No history of penetrating ocular trauma or surgery preceding the initial onset of uveitis	
No clinical or laboratory evidence suggestive of other ocular disease entities	
Bilateral ocular involvement	
Early manifestations	Late manifestations
Evidence of a diffuse choroiditis (with or without anterior uveitis, vitreous inflammatory reaction, or optic disc hyperemia) Focal areas of subretinal fluid or bullous serous retinal detachments	History suggestive of prior presence of findings
	Ocular depigmentation; sunset glow fundus or Sugiura's signs
Equivocal fundus findings: Focal areas of delay in choroidal perfusion, multifocal areas of pinpoint leakage, large placoid areas of hyperfluorescence, pooling within subretinal fluid, and optic nerve staining Diffuse choroidal thickening	Other ocular signs: (a) Nummular chorioretinal depigmented scars (b) Retinal pigment epithelium clumping and/or migration (c) Recurrent or chronic anterior uveitis
Neurological/auditory findings (may have resolved by time of examination); meningismus, tinnitus, or cerebrospinal fluid pleocytosis	
Integumentary finding (not preceding onset of central nervous system or ocular disease), alopecia, or poliosis, or vitiligo	

**Table 2:** Clinical characteristics and management of the cases of VKH.

Case no.	Sex	Age (years)	Stage of presentation	Extraocular manifestation	Presenting VA	Final VA	Medical treatment	Surgical treatment
1	F	54	Acute uveitis	None	HM and HM	6/12 and 6/18	IV steroid, azathioprine	Cataract surgery
2	F	47	Acute uveitis	None	3/60 and 6/18	6/18 and 6/12	Oral steroid, methotrexate	Cataract surgery
3	M	30	Acute uveitis	None	HM and 1/60	6/6 and 6/9	IV steroid, azathioprine, ATT	
4	F	49	Acute uveitis	Integumentary	6/12 and 6/24	6/12 and 6/24	Oral steroid	
5	F	51	Chronic recurrent	None	6/9 and 6/12	6/18 and 6/18	Oral steroid, methotrexate, ATT	
6	F	45	Convalescent	Neurological, integumentary, and auditory	6/18 and 6/9	6/9 and 6/6	Oral steroid, methotrexate	Cataract surgery, glaucoma filtration surgery

M: male; F: female; IV: intravenous; ATT: antitubercular treatment; HM: hand movement. Intravenous corticosteroid as a 500 mg injection of methylprednisolone, twice a day for 3 days. Oral corticosteroid as prednisolone tablet at a dose of 1 mg/kg. Azathioprine tablet at a dose of 1.5 mg/kg/day. Methotrexate tablet at a dose of 7.5 mg once a week along with 5 days of supplementary folic acid 5 mg a week after initial liver function testing.

## Data Availability

Data is available in the text in the form of tables.

## References

[1] Lodhi S., Reddy J., Peram V. (2017). Clinical spectrum and management options in Vogt-Koyanagi-Harada disease. *Clinical Ophthalmology*.

[2] Kharel R., Shah D. N., Chaudhary M. (2016). Presumed Vogt-Koyanagi-Harada (VKH) disease in Nepalese population: a rare entity. *Journal of Clinical Ophthalmology and Research.*.

[3] Liu B., Deng T., Zhu L., Zhong J. (2018). Association of human leukocyte antigen (HLA)-DQ and HLA-DQA1/DQB1 alleles with Vogt-Koyanagi-Harada disease: a systematic review and meta-analysis. *Medicine*.

[4] Agrawal A., Biswas J. (2011). Unilateral Vogt-Koyanagi-Harada disease: report of two cases. *Middle East African Journal of Ophthalmology*.

[5] Usui Y., Goto H. (2009). Presumed Vogt-Koyanagi-Harada disease with unilateral ocular involvement: report of three cases. *Graefe's Archive for Clinical and Experimental Ophthalmology*.

[6] Moorthy R. S., Inomata H., Rao N. A. (1995). Vogt-Koyanagi-Harada syndrome. *Survey of Ophthalmology*.

[7] Neves A., Cardoso A., Almeida M., Campos J., Campos A., Sousa J. P. C. (2015). Unilateral Vogt-Koyanagi-Harada disease: a clinical case report. *Case Reports in Ophthalmology.*.

[8] Forster D. J., Green R. L., Rao N. A. (1991). Unilateral manifestation of the Vogt-Koyanagi-Harada syndrome in a 7-year-old child. *American Journal of Ophthalmology*.

[9] Beniz J., Forster D. J., Lean J. S., Smith R. E., Rao N. A. (1991). Variations in clinical features of the Vogt-Koyanagi-Harada syndrome. *Retina*.

[10] Agarwal M., Radosavljevic A., Patnaik G., Rishi E., Pichi F. (2020). Diagnostic value of optical coherence tomography in the early diagnosis of macular complications in chronic Vogt-Koyanagi-Harada disease. *Ocular Immunology and Inflammation*.

[11] Sakata V. M., da Silva F. T., Hirata C. E., Takahashi W. Y., Costa R. A., Yamamoto J. H. (2014). Choroidal bulging in patients with Vogt-Koyanagi-Harada disease in the non-acute uveitic stage. *Journal of Ophthalmic Inflammation and Infection.*.

[12] Paredes I., Ahmed M., Foster C. (2006). Immunomodulatory therapy for Vogt-Koyanagi-Harada patients as first-line therapy. *Ocular Immunology and Inflammation*.

[13] Dogan B., Erol M. K., Cengiz A. (2016). Vogt-Koyanagi-Harada disease following BCG vaccination and tuberculosis. *Springerplus*.

